# Supplementary material for: Network and state specificity in connectivity‐based predictions of individual behavior
Source: Hum Brain Mapp. 2024 Jun 12;45(8):e26753. doi: 10.1002/hbm.26753 (PMC11167405; doi:10.1002/hbm.26753)
Supplement: Supplementary file 1 — DATA S1: Supporting Information. [file HBM-45-e26753-s001.docx]

Network and State Specificity in Connectivity-Based Predictions of Individual Behavior

# Supplemental Material

Nevena Kraljević^1, 2^, Robert Langner^1, 2^, Vincent Küppers^1, 3^, Federico Raimondo^1, 2^, Kaustubh R. Patil^1, 2^, Simon B. Eickhoff^1, 2^, Veronika I. Müller^2, 1^

# Methods

Overview of references per task:

Table S1: Behavioral Scores used in the prediction

| Domain | “Same” / In-scanner task | “Similar ” / Out-of-scanner task |
| --- | --- | --- |
| Working Memory / WM | N-back (Barch et al., 2013) | List sorting (NIH Toolbox List Sorting Working Memory Test; (*Cognition Measures*, n.d.) |
| Theory of mind / SOCIAL | Labelling of interaction between animated shapes as random or interaction (Castelli et al., 2000; Wheatley et al., 2007) | Compound score “Social Satisfaction” (Babakhanyan et al., 2018) composed of scores for: Friendship, loneliness, emotional support, instrumental support, and perceived rejection all from NIH Toolbox Emotion battery (*Emotion Measures*, n.d.; Salsman et al., 2013). |
| Emotion Recognition / EMO | Face-matching. Adapted by (Hariri et al., 2002) | Penn Emotion Recognition Test (Gur et al., 2002, 2010) |

## Network Delineation

### Network Delineation via Meta-Analysis

As a second approach, to offer feature spaces that are entirely independent from the target, we performed three activation likelihood estimation (ALE) meta-analyses for each of the selected tasks: WM, SOCIAL, and EMO. WM and EMO were based on previous meta-analyses (WM: Rottschy et al., 2012; EMO: Müller, Höhner, et al., 2018), but were extended by including recent publications (findings up to March 2020) and reduced to those tasks that matched the three tasks used in the HCP (i.e. only 2-back vs. 0 back experiments for WM, matching faces > matching shapes for EMO). For SOCIAL we performed our own literature search and coding procedure, following the guidelines for neuroimaging meta-analyses (Müller, Cieslik, et al., 2018) and including experiments that used a theory of mind task using animated shapes and report results of the interaction > random contrast. For each specific task, a meta-analysis was calculated using the ALE algorithm (details about the method see Müller, Cieslik, et al., 2018 and Kogler et al., 2020. From these resulting ALE maps, illustrating spatial convergence across experiments, we extracted all peak coordinates with a minimum distance of 15 mm using FSL. This resulted in three networks from the meta-analyses: MetaWM, MetaSOCIAL, and MetaEMO. The three meta-analytically defined networks will be openly available via the ANIMA-database (Reid et al., 2016; https://anima.fz-juelich.de/).

## Network-based prediction of individual behavior

In addition to PLS, we used Support Vector Regression (SVR), Random Forest, kernel ridge regression algorithms for prediction. Lastly, we performed connectivity-based prediction modelling (CBPM; Finn et al., 2015; Shen et al., 2017) as a popular feature reduction technique with both PLS and kernel ridge as algorithms.

For each algorithm we tuned the hyperparameters in an inner 5x-CV loop. For SVR we ran two different kernels: linear and RBF-kernel. For the linear kernel we tuned the regularization parameter C within [1e-6, 1e-5, 1e-4, 0.0005, 0.001, 0.005], with maximum 2000 iterations. For the RBF-kernel we used the same regularization parameter range, but extended it by [0.01, 0.1, 1, 5, 10]. For the random forest prediction, the number of trees was set to 2000, with mean squared error as the criterion. The number of features was tuned within [0.14, 0.22, 0.33, 0.5, 0.75], with a minimum number of 5 samples required to be at a leaf node. For kernel ridge regression we tuned the lambdas in a range from 0-1000000. In the CBPM feature reduction, feature selection was based on Pearson correlation, retaining features with correlations below the significance threshold of 0.01 and grouping and summing them by positive and negative correlated features. We used the same hyperparameter tuning outlined above with the respective algorithms.

For the significance tests, we used the Nadeau-Bengio machine learning adjusted t-test (Nadeau & Bengio, 1999): $t= \frac{\frac{1}{n}\sum_{j=1}^{n} x_{j}}{\sqrt{(\frac{1}{n} + \frac{n_{test}}{n_{train}})\hat{\sigma}^{2}}}$ . Within each cognitive domain, we first tested effects of state and network by averaging prediction performance of the respective other factors (i.e., averaging across networks and task when testing for state effects, and across state and task when testing for network effects). As domain specificity is an extension of state specificity, we here only averaged across networks for same and similar tasks, respectively. Significant effects (corrected for multiple comparisons) were then further assessed by comparing the respective individual prediction scores between each other. In particular, to assess i) state specificity we compared the prediction performance between states, while keeping network and task constant. That is, we only compared predictions between corresponding networks and tasks (e.g. comparing the prediction performance of “same” WM task score based on FC within Power nodes in resting state to the prediction performance of “same” WM task score based on FC within Power nodes in WM state). To assess ii) network specificity we compared the prediction performance between all networks, while keeping state and task constant (e.g. comparison of prediction performance of “same” WM score in resting state WM networks compared to “same” WM score in resting state in EMO networks). To assess iii) domain specificity we compared the prediction performance of “same” and “similar” task scores, while keeping state and network constant (e.g. comparison of prediction performance of “same” WM task score based on Power nodes in WM state to prediction performance of “similar” WM task score based on Power nodes in WM state).

# Results

| WM | SOCIAL | EMO |
| --- | --- | --- |
| 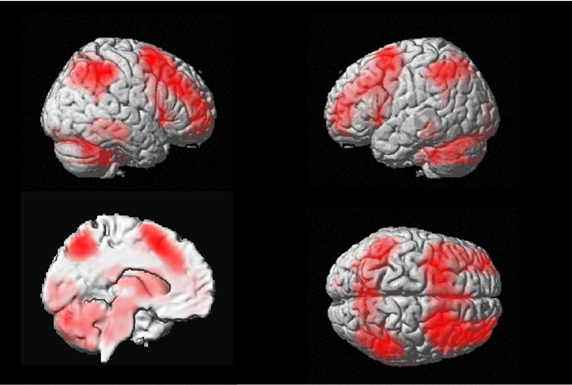  Figure S1. Group-level activation map of WM-task (p < 0.05 (cluster-level FWE-corrected threshold 0.05, cluster-forming threshold p < 0.001) | 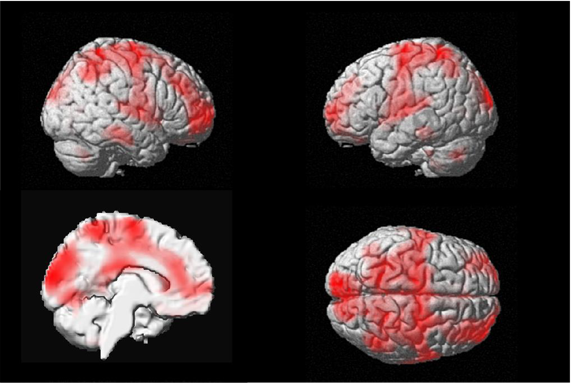  Figure S2. Group-level activation map of SOCIAL-task (cluster-level FWE-corrected threshold 0.05, cluster-forming threshold p < 0.001) | 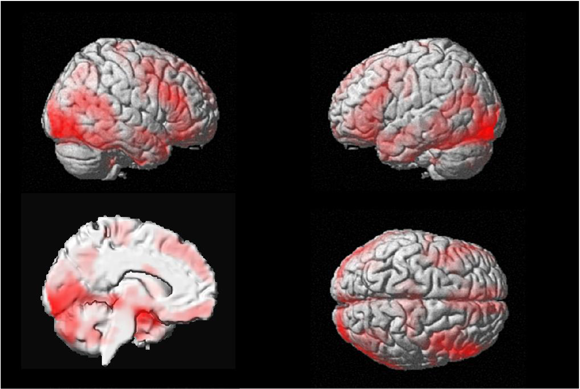  Figure S3. Group-level activation map of EMO-task (cluster-level FWE-corrected threshold 0.05, cluster-forming threshold p < 0.001) |
| 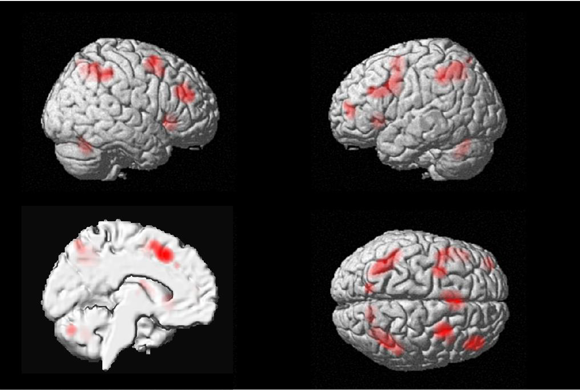  Figure S4. Activation likelihood estimation map of WM meta-analysis (cluster-level FWE-corrected threshold 0.05, cluster-forming threshold p < 0.001). | 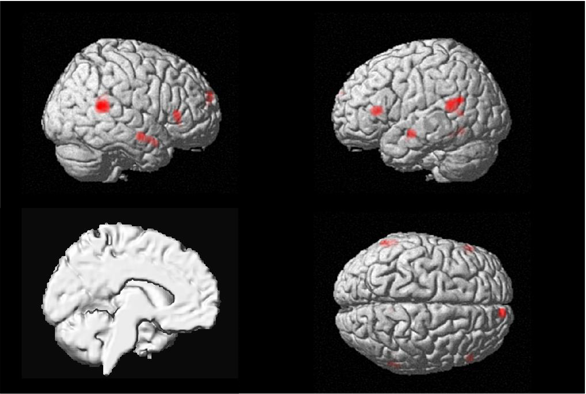  Figure S5. Activation likelihood estimation map of SOCIAL meta-analysis (cluster-level FWE-corrected threshold 0.05, cluster-forming threshold p < 0.001). | 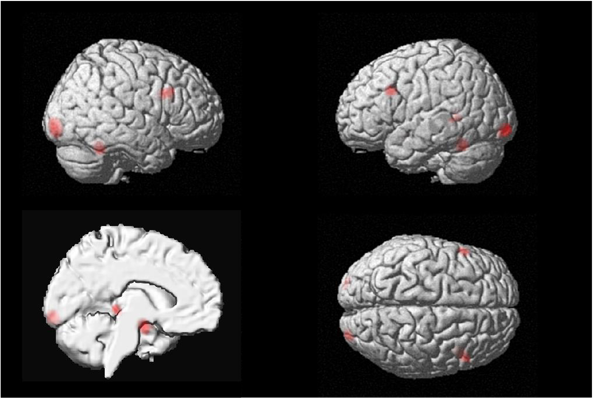  Figure S6. Activation likelihood estimation map of EMO meta-analysis (cluster-level FWE-corrected threshold 0.05, cluster-forming threshold p < 0.001). |

Table S2. List of peak coordinates for WM-NW, extracted with a minimum distance of 15 mm (from Fig. S1)

| **Node** | **X** | **Y** | **Z** | **Brain Structure** |
| --- | --- | --- | --- | --- |
| 1 | 34 | -58 | -32 | R Cerebellum Crus I |
| 2 | 46 | -46 | 46 | R Intraparietal Suclus |
| 3 | -30 | -60 | -32 | L Cerebellum VI |
| 4 | -6 | 18 | 48 | L Paracingulate Gyrus |
| 5 | 32 | 6 | 58 | R Middle Frontal Gyrus |
| 6 | -28 | 6 | 54 | L Middle Frontal Gyrus |
| 7 | -44 | -52 | 46 | L Intraparietal Sulcus |
| 8 | 40 | 34 | 28 | R Middle Frontal Gyrus |
| 9 | -8 | -64 | 50 | L Superior Parietal Lobule |
| 10 | 8 | -68 | 54 | R Precuneous Cortex |
| 11 | 34 | 22 | 4 | R anterior Insular Cortex |
| 12 | -32 | 50 | 16 | L Frontal Pole |
| 13 | -32 | 20 | 0 | L anterior Insular Cortex |
| 14 | -44 | 26 | 34 | L Middle Frontal Gyrus |
| 15 | 38 | -60 | -48 | R Cerebellum Crus II |
| 16 | 12 | -76 | -24 | R Cerebellum Crus I |
| 17 | 38 | 48 | 18 | R Frontal Pole |
| 18 | -8 | -80 | -26 | L Cerebellum Crus I |
| 19 | -16 | 8 | 12 | L Caudate |
| 20 | 18 | 10 | 16 | R Caudate |
| 21 | 58 | -30 | -14 | R posterior Middle Temporal Gyrus |
| 22 | -8 | -58 | -54 | L Cerebellum IX |
| 23 | 52 | 10 | 16 | R Inferior Frontal Gyrus |
| 24 | 24 | 46 | -14 | R Frontal Pole |
| 25 | -12 | -92 | 2 | L Occipital Pole |
| 26 | 10 | 2 | 6 | R Thalamus |
| 27 | 0 | -50 | -18 | Cerebellum I-IV |
| 28 | 0 | -30 | -4 | L Thalamus |
| 29 | 8 | -58 | -54 | R Cerebellum IX |
| 30 | 48 | 6 | 30 | R Precentral Gyrus |
| 31 | -24 | 50 | -12 | L Frontal Pole |
| 32 | 2 | -12 | 16 | R Thalamus |
| 33 | 0 | -62 | -36 | Cerebellum Vermis VIIIb |
| 34 | -56 | -36 | -14 | L Middle Temporal Gyrus |
| 35 | 2 | 12 | 24 | Cingulate Gyrus |
| 36 | -2 | -32 | 24 | R Midcingulate Gyrus |
| 37 | -44 | -50 | 20 | L Angular Gyrus |
| 38 | 28 | -58 | 66 | R Superior Lateral Occipital Cortex |
| 39 | -14 | -26 | -32 | Brain Stem |
| 40 | 20 | -28 | 14 | R Thalamus |
| 41 | 20 | -96 | -14 | R Occipital Pole |
| 42 | 34 | -90 | -18 | R Inferior Lateral Occipital Cortex |
| 43 | 16 | 28 | -22 | R Frontal Orbital Cortex |
| 44 | 24 | -20 | -6 | R Hippocampus |
| 45 | -22 | -58 | 0 | L Lingual Gyrus |

Table S3. List of peak coordinates for WM-Meta, extracted with a minimum distance of 15 mm (from Fig. S4)

| **Node** | **X** | **Y** | **Z** | **Brain Structure** |
| --- | --- | --- | --- | --- |
| 1 | -46 | 6 | 36 | L Middle Frontal Gyrus |
| 2 | -28 | 2 | 54 | L Middle Frontal Gyrus |
| 3 | -46 | 26 | 28 | L Middle Frontal Gyrus |
| 4 | -34 | -54 | 48 | L Intraparietal Suclus |
| 5 | 42 | -46 | 44 | R Intraparietal Sulcus |
| 6 | 10 | -66 | 52 | R Precuneous Cortex |
| 7 | -2 | 18 | 48 | L Pre-supplementary motor area |
| 8 | -2 | 32 | 38 | L Paracingulate Gyrus |
| 9 | 30 | 8 | 56 | R Middle Frontal Gyrus |
| 10 | 32 | 24 | -2 | R anterior insular cortex |
| 11 | 44 | 34 | 26 | R Middle Frontal Gyrus |
| 12 | -32 | -60 | -34 | L Cerebellum Crus I |
| 13 | 30 | -60 | -30 | R Cerebellum IV |
| 14 | -32 | 22 | 0 | L anteior insular cortex |
| 15 | -38 | 50 | 8 | L Frontal Pole |
| 16 | 10 | -76 | -24 | R Cerebellum IV |
| 17 | -8 | -76 | -28 | L Cerebellum Crus I |
| 18 | -12 | -68 | 60 | L Superior Parietal Lobule |
| 19 | -16 | -2 | 16 | L Caudate |

Table S4. List of peak coordinates for SOCIAL -NW, extracted with a minimum distance of 15 mm (from Fig. S2)

| **Node** | **X** | **Y** | **Z** | **Brain Structure** |
| --- | --- | --- | --- | --- |
| 1 | -12 | -94 | 18 | L Occipital Pole |
| 2 | -22 | -52 | 64 | L Superior Parietal Lobule |
| 3 | 22 | -50 | 68 | R Superior Parietal Lobule |
| 4 | 18 | -86 | 24 | R Superior Lateral Occipital Cortex |
| 5 | -10 | -80 | 34 | L Cuneal Cortex |
| 6 | -18 | -12 | 70 | L Precentral Gyrus |
| 7 | 10 | -80 | -6 | R Lingual Gyrus |
| 8 | 12 | -74 | 38 | R Precuneous Cortex |
| 9 | 16 | -10 | 74 | R Superior Frontal Gyrus |
| 10 | 26 | -44 | 10 | R Precuneous Cortex |
| 11 | 2 | -24 | 30 | R Posterior Cingulate Gyrus |
| 12 | 28 | 66 | 2 | R Frontal Pole |
| 13 | 48 | -56 | 46 | R Inferior Parietal Lobules |
| 14 | 40 | 54 | -6 | R Lateral Frontal Orbital Cortex |
| 15 | -26 | 68 | 4 | L Frontal Pole |
| 16 | 0 | 16 | 10 | Septum |
| 17 | 40 | 48 | 10 | R Frontal Pole |
| 18 | 50 | -2 | -2 | R Planum Polare |
| 19 | -20 | -26 | 76 | L Precentral Gyrus |
| 20 | -2 | -10 | 70 | L Supplementary Motor Area |
| 21 | 2 | 40 | 16 | R Anterior Cingulate Gyrus |
| 22 | -46 | -10 | 60 | L Precentral Gyrus |
| 23 | -22 | -76 | 4 | L Intracalcarine Cortex |
| 24 | 42 | 38 | 32 | R Middle Frontal Gyrus |
| 25 | -12 | 26 | 6 | L Caudate |
| 26 | -48 | -2 | -4 | L Planum Polare |
| 27 | -8 | -76 | -4 | L Lingual Gyrus |
| 28 | 38 | 14 | 8 | R Frontal Operculum Cortex |
| 29 | 4 | 46 | 2 | R Anterior Cingulate Gyrus |
| 30 | -42 | -26 | 62 | L Postcentral Gyrus |
| 31 | 48 | -8 | 52 | R Precentral Gyrus |
| 32 | 20 | -26 | 76 | R Precentral Gyrus |
| 33 | -62 | 2 | 20 | R Precentral Gyrus |
| 34 | -46 | -36 | 16 | L Inferior Parietal Lobule |
| 35 | 2 | 4 | 50 | R Supplementary Motor Area |
| 36 | 14 | 26 | 8 | R Caudate |
| 37 | 42 | -22 | 62 | R Postcentral Gyrus |
| 38 | 20 | -30 | 24 | R Thalamus |
| 39 | 2 | -30 | 50 | R Precentral Gyrus |
| 40 | -42 | -60 | 48 | L Intraparietal Sulcus |
| 41 | -8 | -10 | 52 | L Supplementary Motor Area |
| 42 | -26 | 46 | -12 | L Medial Frontal Obital Cortex |
| 43 | 66 | -20 | 8 | R posterior Superior Temporal Gyrus |
| 44 | -42 | 12 | -8 | R Anterior Insular Cortex |
| 45 | 14 | -16 | 46 | R Precentral Gyrus |
| 46 | -38 | -14 | 24 | L Parietal Opercular Cotex |
| 47 | 60 | -2 | 38 | R Precentral Gyrus |
| 48 | -36 | 12 | 10 | L Anterior Insular Cortex |
| 49 | -28 | 44 | 36 | L Frontal Pole |
| 50 | 36 | -10 | 20 | R Parietal Opercular Cortex |
| 51 | 30 | 22 | 60 | R Middle Frontal Gyrus |
| 52 | -12 | 18 | -16 | L Medial Frontal Obital Cortex |
| 53 | 40 | -66 | 8 | R inferior Lateral Occipital Cortex |
| 54 | 12 | 48 | -26 | R medial frontal orbital cortex |
| 55 | -14 | 32 | -22 | L Frontal Orbital Cortex |
| 56 | 2 | -70 | -20 | Cerebellum Vermis VI |
| 57 | 44 | -36 | 34 | R Intraparietal Sulcus |
| 58 | 44 | -32 | 18 | R Inferior Parietal Lobule |
| 59 | -32 | -52 | 30 | L Intraparietal Sulcus |
| 60 | -46 | 32 | 38 | L Middle Frontal Gyrus |
| 61 | 62 | -26 | -16 | R posterior Middle Temporal Gyrus |
| 62 | -46 | -66 | -38 | L Cerebellum Crus I |
| 63 | -64 | -30 | -14 | L Posterior Middle Temporal Gyrus |
| 64 | -26 | -48 | -50 | L Cerebellum VIIIb |
| 65 | -34 | -34 | -38 | L Cerebellum VI |
| 66 | 48 | -62 | -38 | R Cerebellum Crus I |

Table S5. List of peak coordinates for SOCIAL-Meta, extracted with a minimum distance of 15 mm (from Fig. S5)

| **Node** | **X** | **Y** | **Z** | **Brain Structure** |
| --- | --- | --- | --- | --- |
| 1 | 58 | -48 | 14 | R Inferior Parietal Lobule |
| 2 | -58 | -46 | 16 | L Supramarginal Gyrus |
| 3 | 62 | -8 | -16 | R Middle Temporal Gyrus |
| 4 | 54 | 6 | -22 | R Temporal Pole |
| 5 | 10 | 62 | 22 | R Frontal Pole |
| 6 | -44 | -58 | -10 | L Mid Fusiform Gyrus |
| 7 | 54 | 28 | 6 | R Inferior Frontal Gyrus |
| 8 | -54 | 26 | 10 | L Inferior Frontal Gyrus |
| 9 | 8 | -48 | 50 | R Precuneous Cortex |
| 10 | 8 | -54 | 36 | R Precuneous Cortex |
| 11 | -60 | -8 | -14 | R Middle Temporal Gyrus |

Table S6. List of peak coordinates for EMO-NW, extracted with a minimum distance of 15 mm (from Fig. S3)

| **Node** | **X** | **Y** | **Z** | **Brain Structure** |
| --- | --- | --- | --- | --- |
| 1 | 24 | -96 | -4 | R Occipital Pole |
| 2 | 42 | -48 | -20 | R Mid Fusiform Gyrus |
| 3 | -20 | -94 | -12 | L Occipital Pole |
| 4 | 38 | -72 | -14 | R Posterior Fusiform Gyrus |
| 5 | 18 | -4 | -16 | R Amygdala |
| 6 | -18 | -4 | -18 | L Amygdala |
| 7 | -34 | -86 | -12 | L inferior lateral Occipital Cortex |
| 8 | -40 | -54 | -20 | L Mid Fusiform Gyrus |
| 9 | 44 | 18 | 24 | R Inferior Frontal Gyrus |
| 10 | -4 | -82 | 2 | L Intracalcarine Cortex |
| 11 | 14 | -32 | -2 | R Thalamus |
| 12 | -8 | -76 | -38 | L Cerebellum Crus II |
| 13 | 34 | 34 | -14 | R Frontal Pole |
| 14 | 32 | -6 | -38 | R Parahippocampal Gyrus |
| 15 | -40 | 18 | 26 | L Middle Frontal Gyrus |
| 16 | 48 | -64 | 18 | R Superior Lateral Occipital Cortex |
| 17 | -10 | -32 | -2 | L Thalamus |
| 18 | -34 | -10 | -32 | L Parahippocampus |
| 19 | 14 | -70 | 10 | R Intracalcarine Cortex |
| 20 | 50 | -42 | 14 | R Posterior Superior Termporal Sulcus |
| 21 | -24 | -24 | -8 | L Hippocampus |
| 22 | -36 | 30 | -16 | L Frontal Orbital Cortex |
| 23 | -2 | -2 | -16 | L Hypothalamus |
| 24 | 20 | -38 | -44 | R Cerebellum X |
| 25 | 0 | -52 | -36 | Cerebellum Vermis IX |
| 26 | 22 | -52 | 4 | R Lingual Gyrus |
| 27 | -20 | -36 | -44 | L Cerebellum X |
| 28 | -50 | -72 | 18 | L Superior Lateral Occipital Cortex |
| 29 | 50 | -10 | -12 | R Superior Temporal Gyrus |
| 30 | 62 | -52 | 12 | R Middle Temporal Gyrus |
| 31 | 60 | -42 | -4 | R Middle Temporal Gyrus |
| 32 | 10 | -78 | -38 | R Cerebellum Crus II |
| 33 | 2 | 52 | -14 | R Frontal Medial Cortex |
| 34 | 46 | 2 | 54 | R Middle Frontal Gyrus |
| 35 | -52 | -46 | 12 | L Posterior Superior Termporal Sulcus |
| 36 | 34 | -56 | 44 | R Intraparietal Sulcus |
| 37 | -44 | 40 | -2 | L Frontal Pole |
| 38 | -60 | -40 | -8 | L Middle Temporal Gyrus |
| 39 | 4 | -60 | 40 | R Precuneus |
| 40 | 0 | 4 | 28 | Midcingulate Cortex |
| 41 | 36 | -74 | 24 | R superior lateral Occipital Cortex |
| 42 | 4 | 58 | 32 | R Frontal Pole |
| 43 | 2 | 38 | 50 | R medial superior Frontal Gyrus |
| 44 | 52 | 28 | -6 | R Inferior Frontal Gyrus |
| 45 | -30 | -68 | -48 | L Cerebellum VIIb |
| 46 | -52 | -10 | -12 | L Superior Temporal Gyrus |
| 47 | 22 | -68 | 24 | R Parieto-occipital sulcus |
| 48 | 6 | -12 | 6 | R Thalamus |
| 49 | 24 | -32 | -20 | R Parahippocampal Gyrus |
| 50 | -40 | 2 | 54 | L Middle Frontal Gyrus |
| 51 | 28 | -24 | 60 | R Precentral Gyrus |
| 52 | -50 | 12 | 46 | L Middle Frontal Gyrus |
| 53 | 22 | 0 | 6 | R Putamen |
| 54 | 34 | 20 | 54 | R Middle Frontal Gyrus |
| 55 | 8 | -96 | 26 | R Occipital Pole |
| 56 | -4 | -22 | 58 | L Precentral Gyrus |
| 57 | 16 | -26 | 72 | R Precentral Gyrus |
| 58 | 2 | 4 | -2 | R Basal Forebrain |
| 59 | -18 | -92 | -28 | L Cerebellum Crus I |
| 60 | 8 | 6 | 12 | R Caudate |
| 61 | 0 | -86 | 36 | Cuneal Cortex |
| 62 | -14 | -26 | 72 | L Precentral Gyrus |
| 63 | 6 | -40 | 68 | R Postcentral Gyrus |
| 64 | -64 | -6 | 28 | L Postcentral Gyrus |
| 65 | -12 | 10 | 4 | L Caudate |
| 66 | 66 | -2 | 28 | R Postcentral Gyrus |
| 67 | -50 | -10 | 32 | L Precentral Gyrus |
| 68 | -50 | -28 | -2 | L Posterior Superior Temporal Gyrus |
| 69 | -38 | 18 | 58 | L Middle Frontal Gyrus |
| 70 | 18 | -12 | 20 | R Caudate |
| 71 | -38 | -18 | 40 | L Postcentral Gyrus |
| 72 | 12 | -10 | 78 | R Superior Frontal Gyrus |
| 73 | 2 | 12 | 70 | R Pre-Supplementary Motor Area |
| 74 | -12 | -40 | 66 | L Postcentral Gyrus |
| 75 | 30 | -22 | 12 | R Posterior Insula |
| 76 | -14 | -2 | 16 | L Caudate |
| 77 | -18 | 30 | 60 | L Superior Frontal Gyrus |
| 78 | -30 | -80 | 26 | L Superior Lateral Occipital Cortex |
| 79 | -58 | -12 | 48 | L Postcentral Gyrus |
| 80 | 34 | -68 | -50 | R Cerebellum VIIb |
| 81 | -60 | -10 | -36 | L Anterior Inferior Temporal Gyrus |
| 82 | -50 | -68 | 46 | L Superior Lateral Occipital Cortex |
| 83 | -32 | -60 | 44 | L Intraparietal Sulcus |

Table S7. List of peak coordinates for EMO-Meta, extracted with a minimum distance of 15 mm (from Fig. S6)

| **Node** | **X** | **Y** | **Z** | **Brain Structure** |
| --- | --- | --- | --- | --- |
| 1 | 20 | -4 | -18 | R Amygdala |
| 2 | 28 | -94 | -6 | R Occipital Pole |
| 3 | -22 | -6 | -14 | L Amygdala |
| 4 | -22 | -96 | -6 | L Occipital Pole |
| 5 | 42 | 12 | 28 | R Inferior Frontal Gyrus |
| 6 | -42 | -54 | -22 | L Mid Fusiform Gyrus |
| 7 | 40 | -50 | -26 | R Mid Fusiform Gyrus |
| 8 | -18 | -32 | -2 | L Thalamus |
| 9 | -50 | -48 | 4 | L Posterior Superior Termporal Sulcus |
| 10 | -54 | 18 | 32 | L Middle Frontal Gyrus |

| 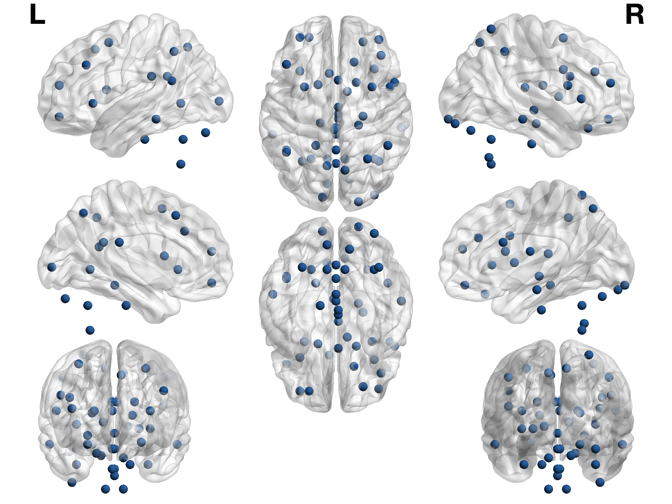  Figure S7. WM Network Nodes. | 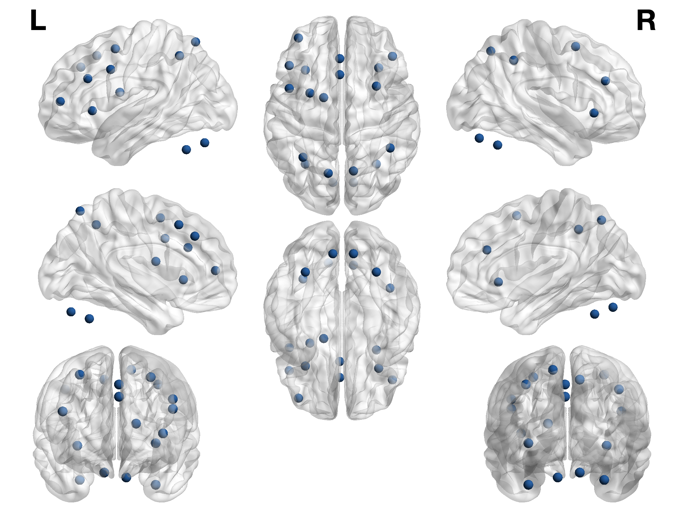  Figure S8. WM Meta-Analysis Network Nodes. |
| --- | --- |

| 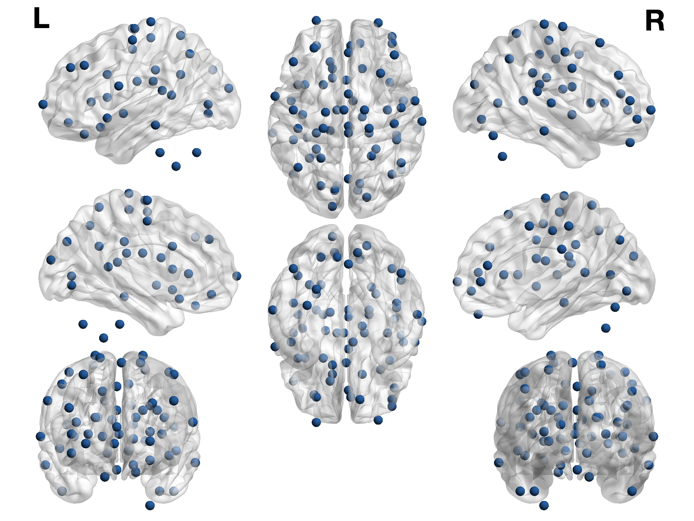  Figure S9. SOCIAL Network Nodes. | 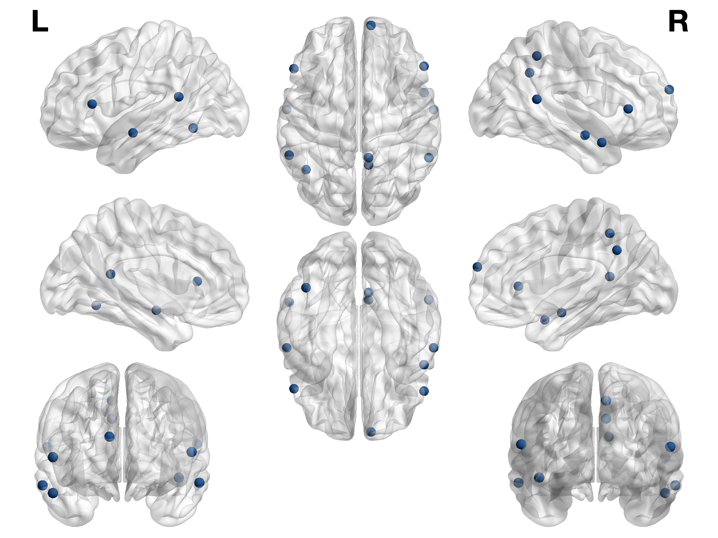  Figure S10. SOCIAL Meta-Analysis Network Nodes |
| --- | --- |

| 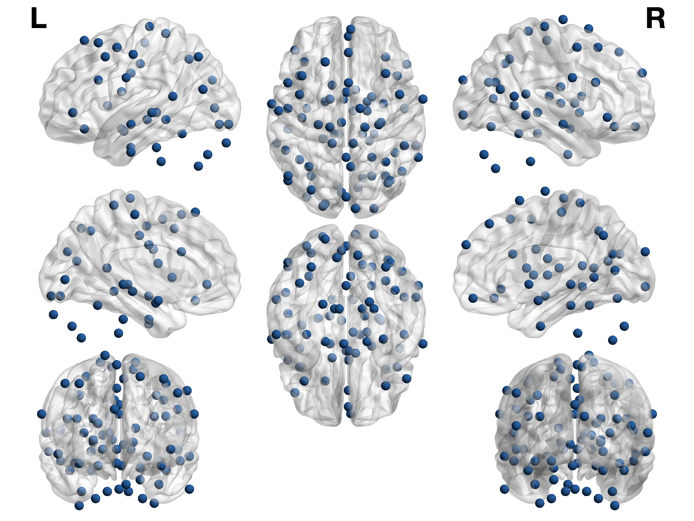  Figure S11. EMO Network Nodes | 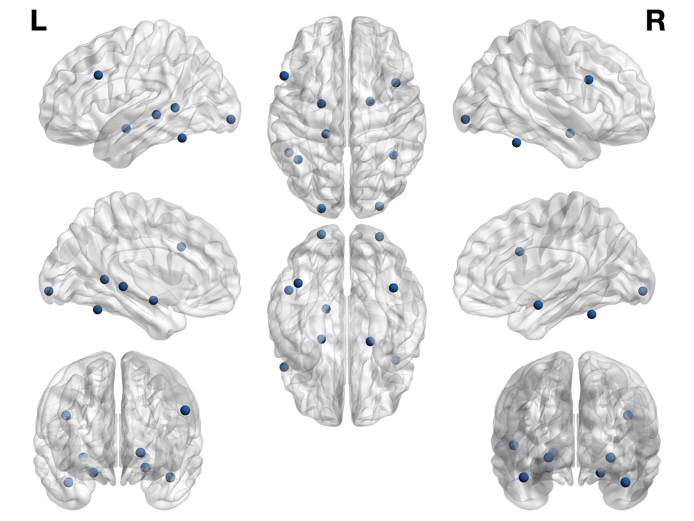  Figure S12. EMO Meta-Analysis Network Nodes |
| --- | --- |

| 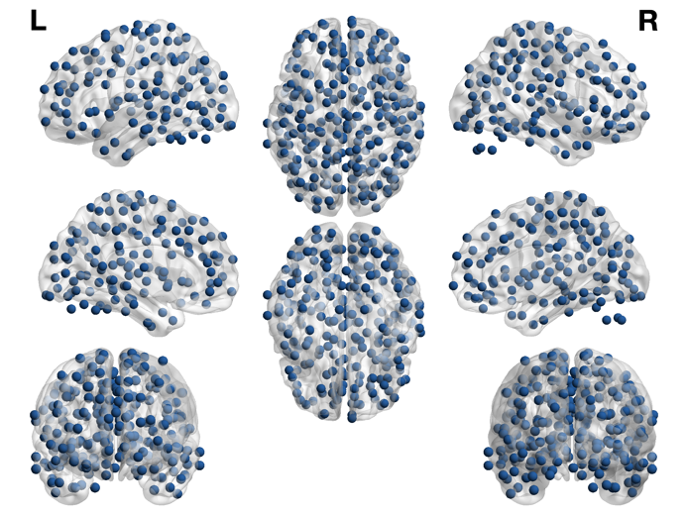  Figure S13. Whole-brain Power nodes. |
| --- |


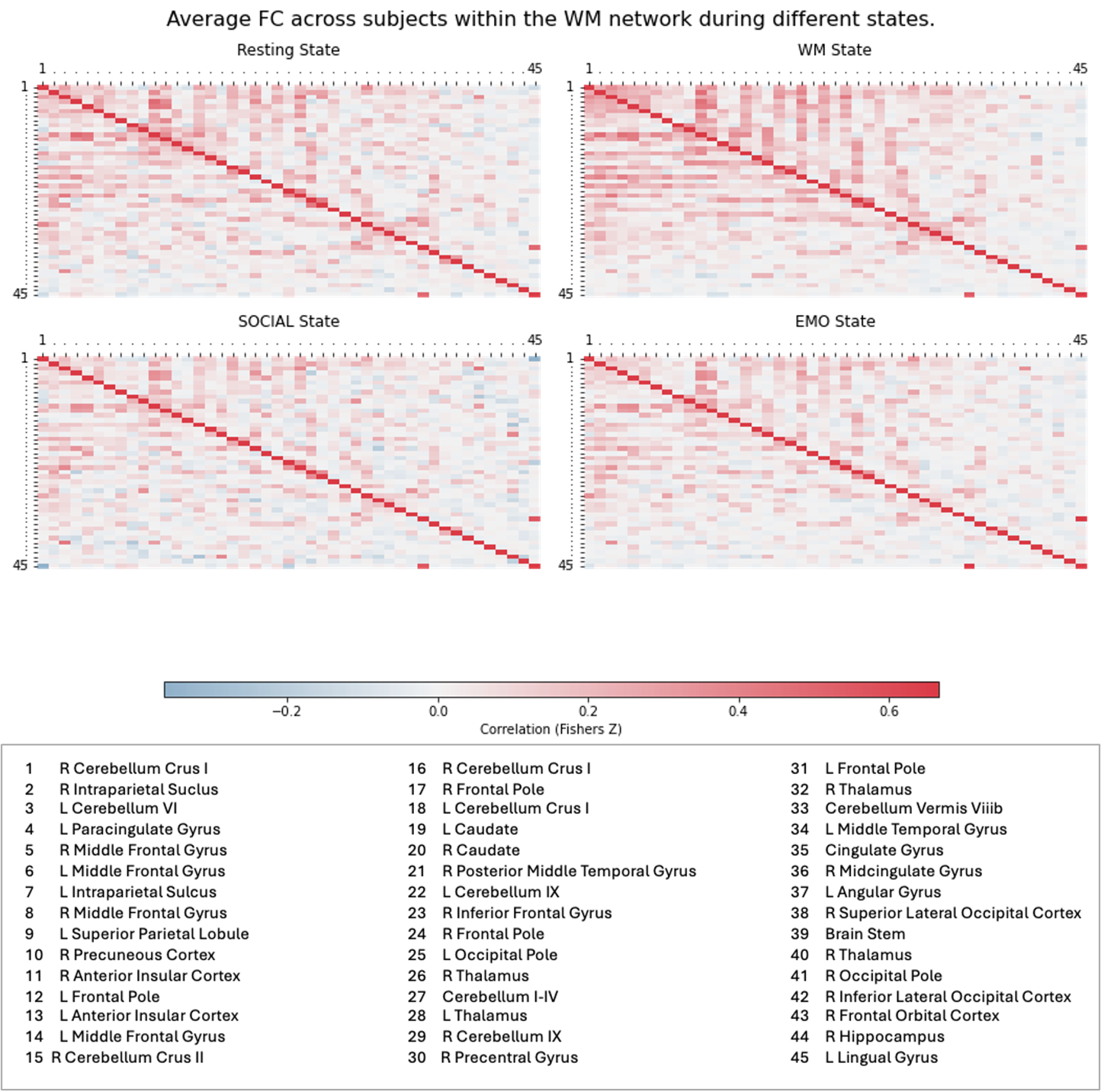


Figure S14) Heatmap of FC within the WM network averaged across participants in the four different states. FC reflects the Fisher Z- transformed Pearson correlation coefficients between all network nodes. Anatomical labels and coordinates of the nodes can be found in table S2-, node numbers are in the same order as in the table.


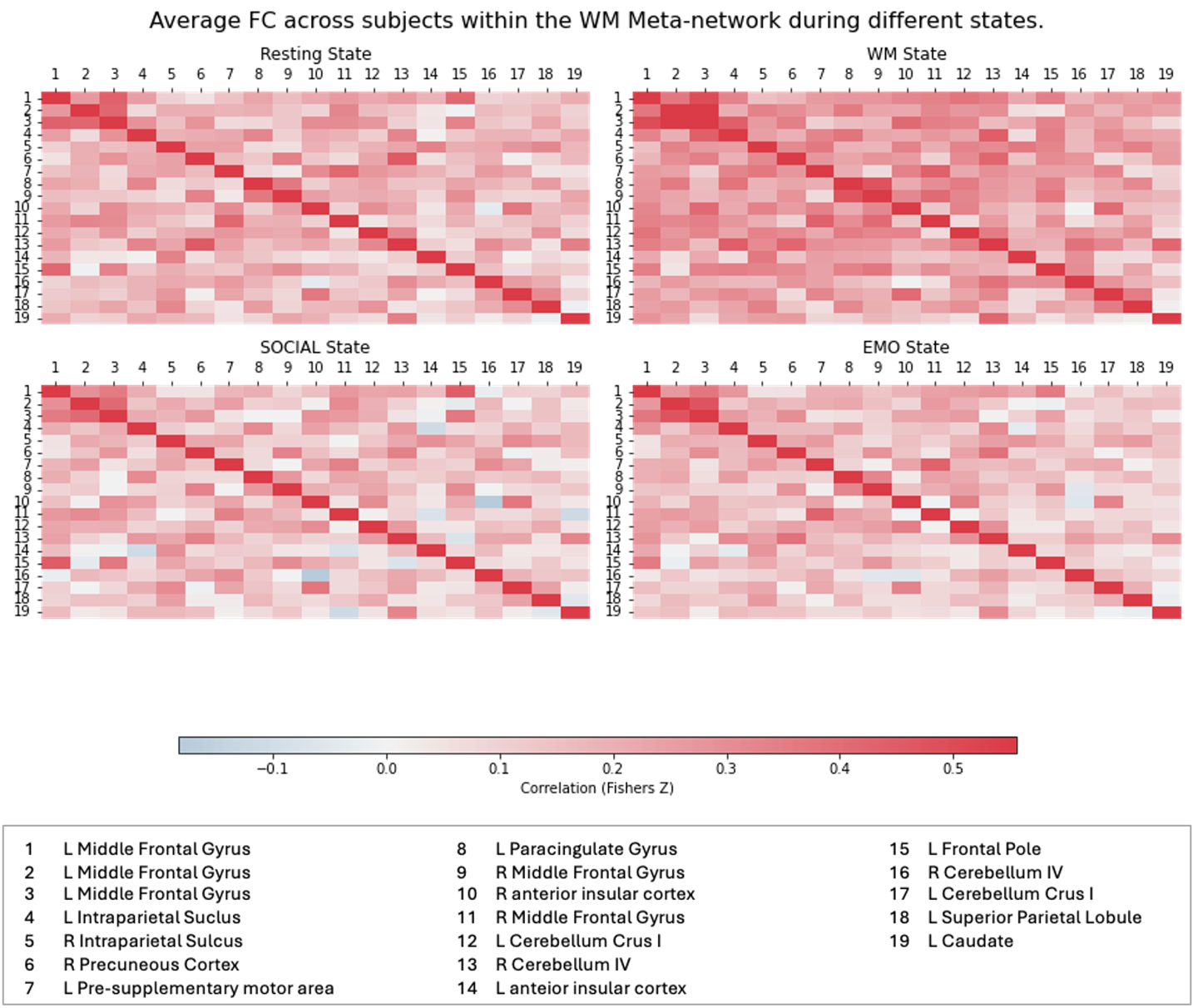


Figure S15) Heatmap of FC within the WM-meta network averaged across participants during the four different states. FC reflects the Fisher Z- transformed Pearson correlation coefficients between all network nodes. Anatomical labels and coordinates of the nodes can be found in table S2-, node number correspond to the node number in the table.


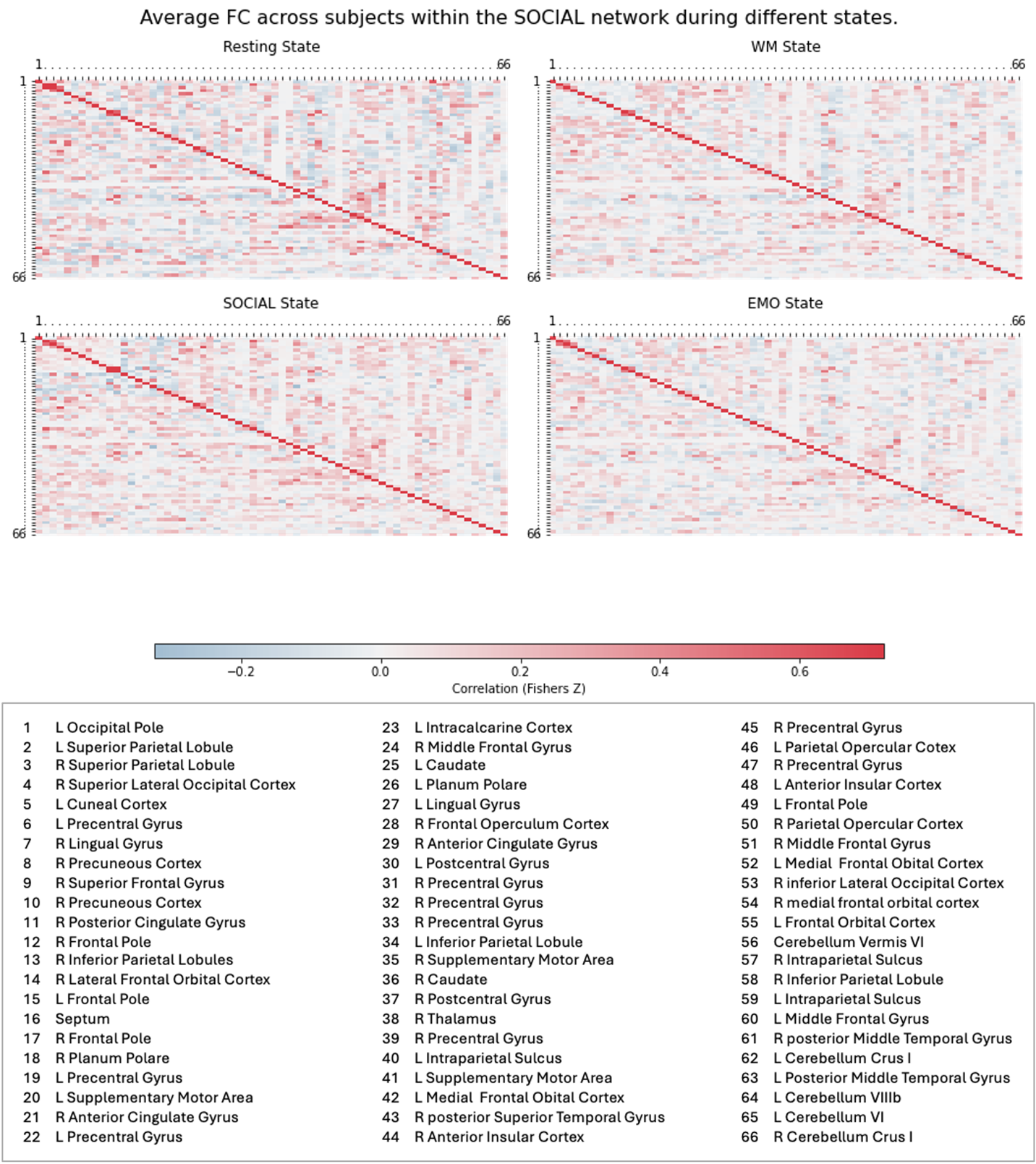


Figure S16) Heatmap of FC within the SOCIAL network averaged across participants during the four different states.. FC reflects the Fisher Z- transformed Pearson correlation coefficients between all network nodes. Anatomical labels and coordinates of the nodes can be found in table S3, node number correspond to the node number in the table.


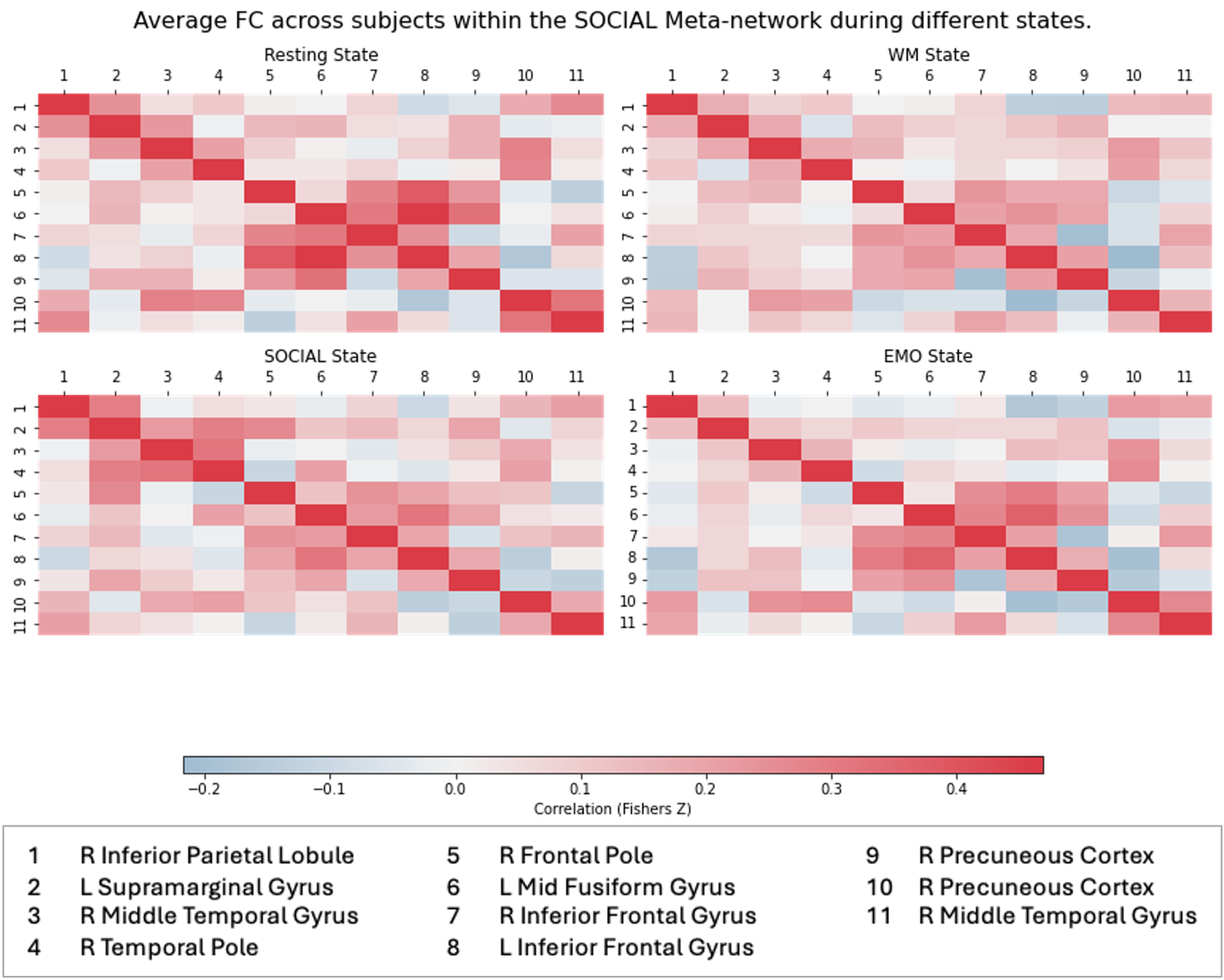


Figure S17) Heatmap of FC within the SOCIAL-meta network averaged across participants during the four different states.. FC reflects the Fisher Z- transformed Pearson correlation coefficients between all network nodes. Anatomical labels and coordinates of the nodes can be found in table S3, node number correspond to the node number in the table.


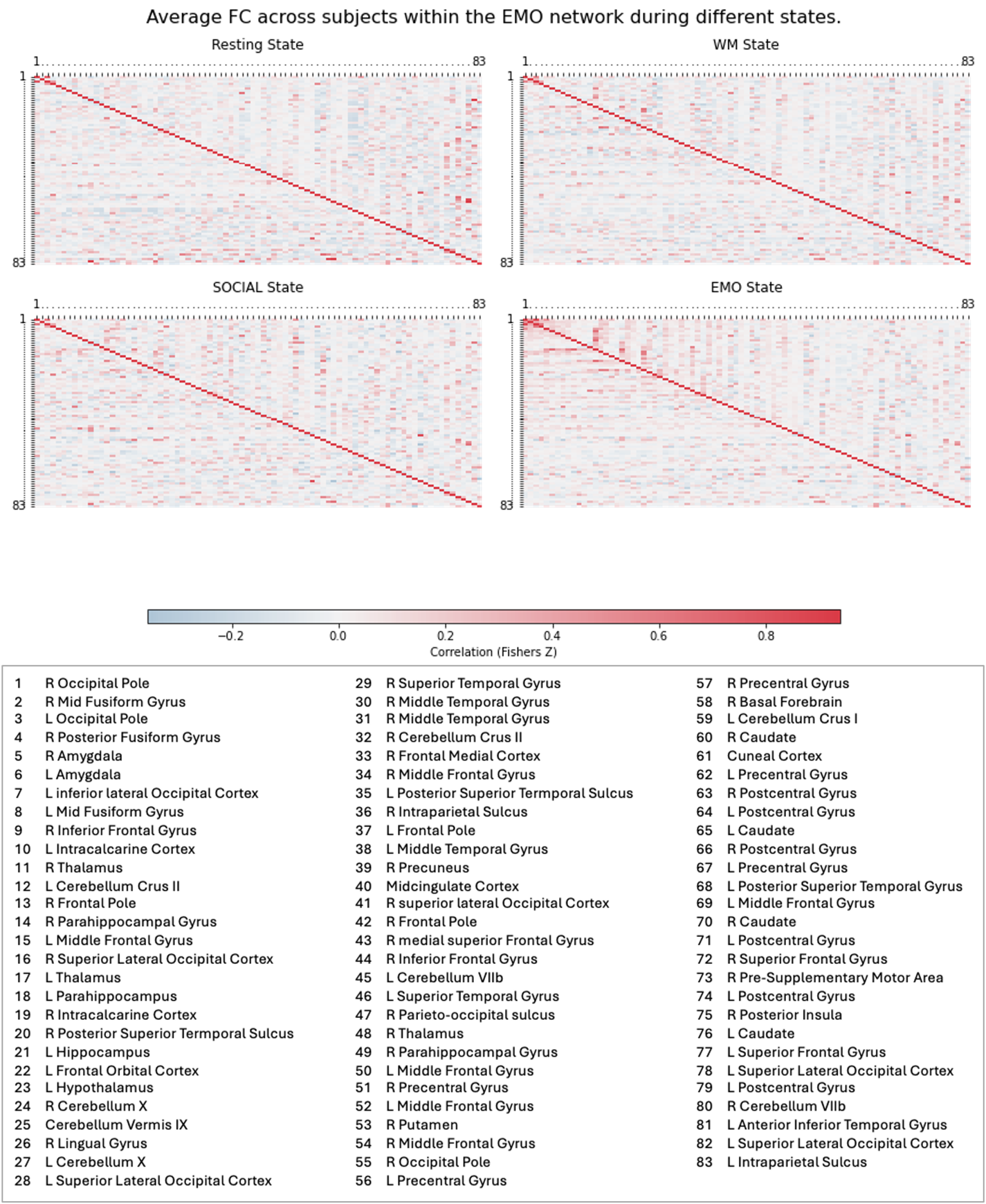


Figure S18) Heatmap of FC within the EMO network averaged across participants during the four different states.. FC reflects the Fisher Z- transformed Pearson correlation coefficients between all network nodes. Anatomical labels and coordinates of the nodes can be found in table S4, node number correspond to the node number in the table.


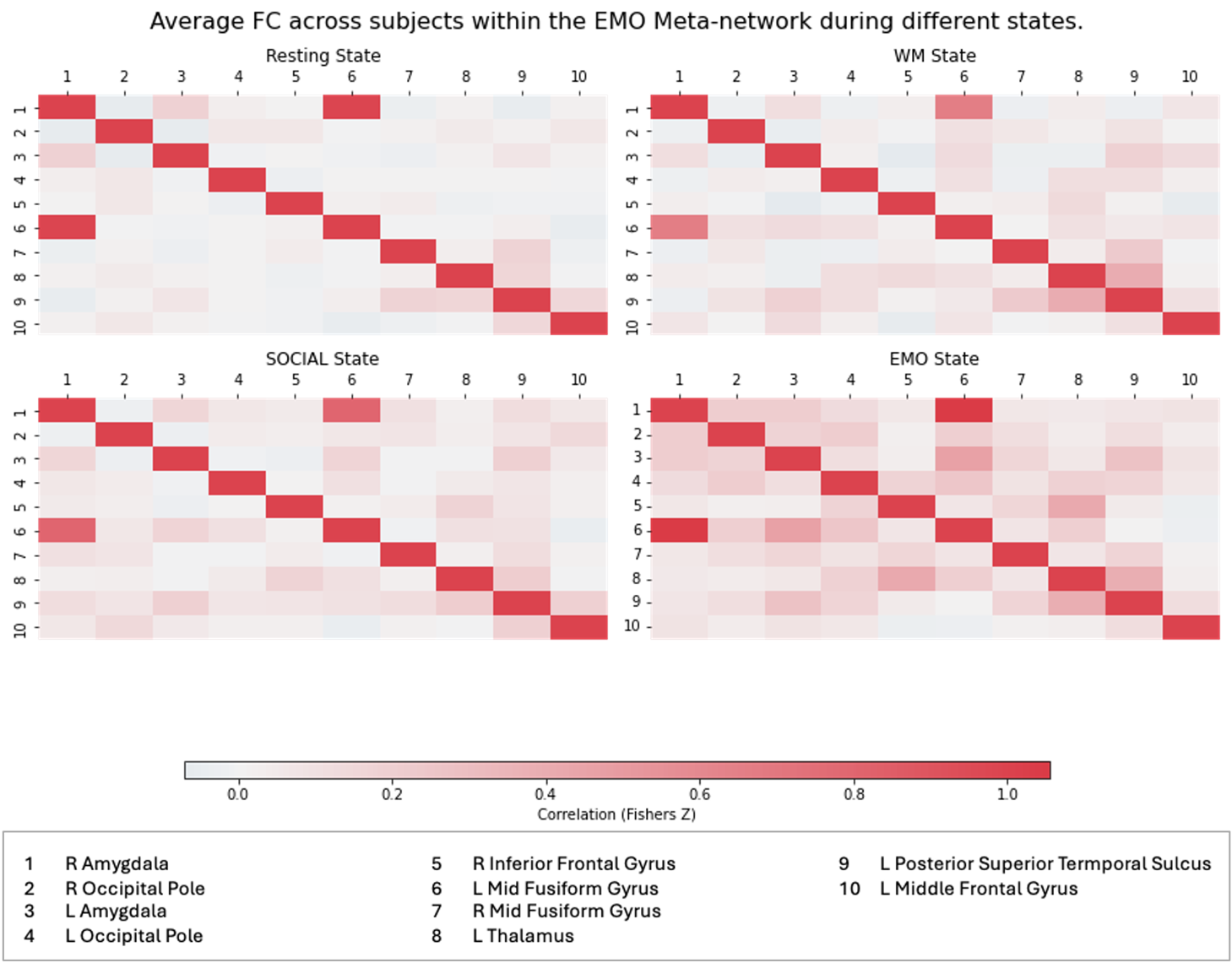


Figure S19) Heatmap of FC within the EMO-meta network averaged across participants during the four different states.. FC reflects the Fisher Z- transformed Pearson correlation coefficients between all network nodes. Anatomical labels and coordinates of the nodes can be found in table S4, node number correspond to the node number in the table.


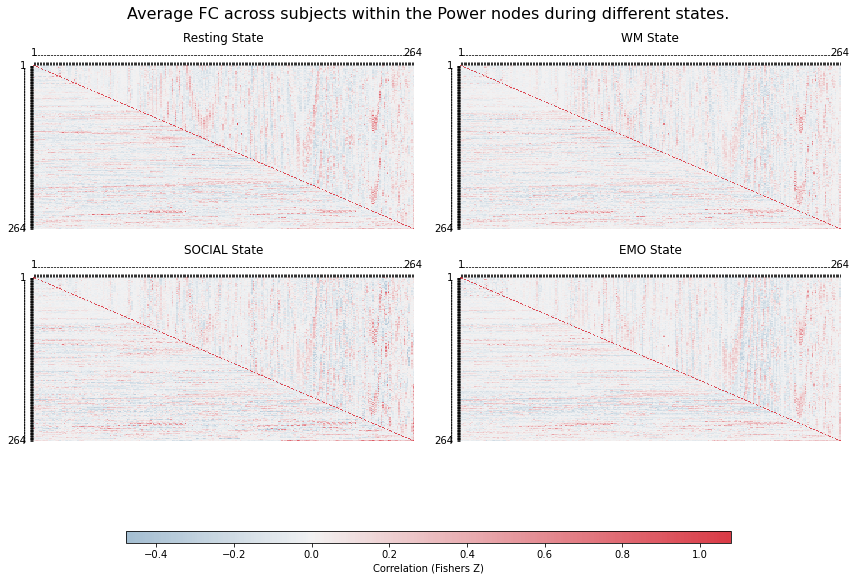


Figure S20) Heatmap of FC within the Power nodes during the four different states averaged across participants. FC reflects the Fisher Z- transformed Pearson correlation coefficients between all network nodes.

| 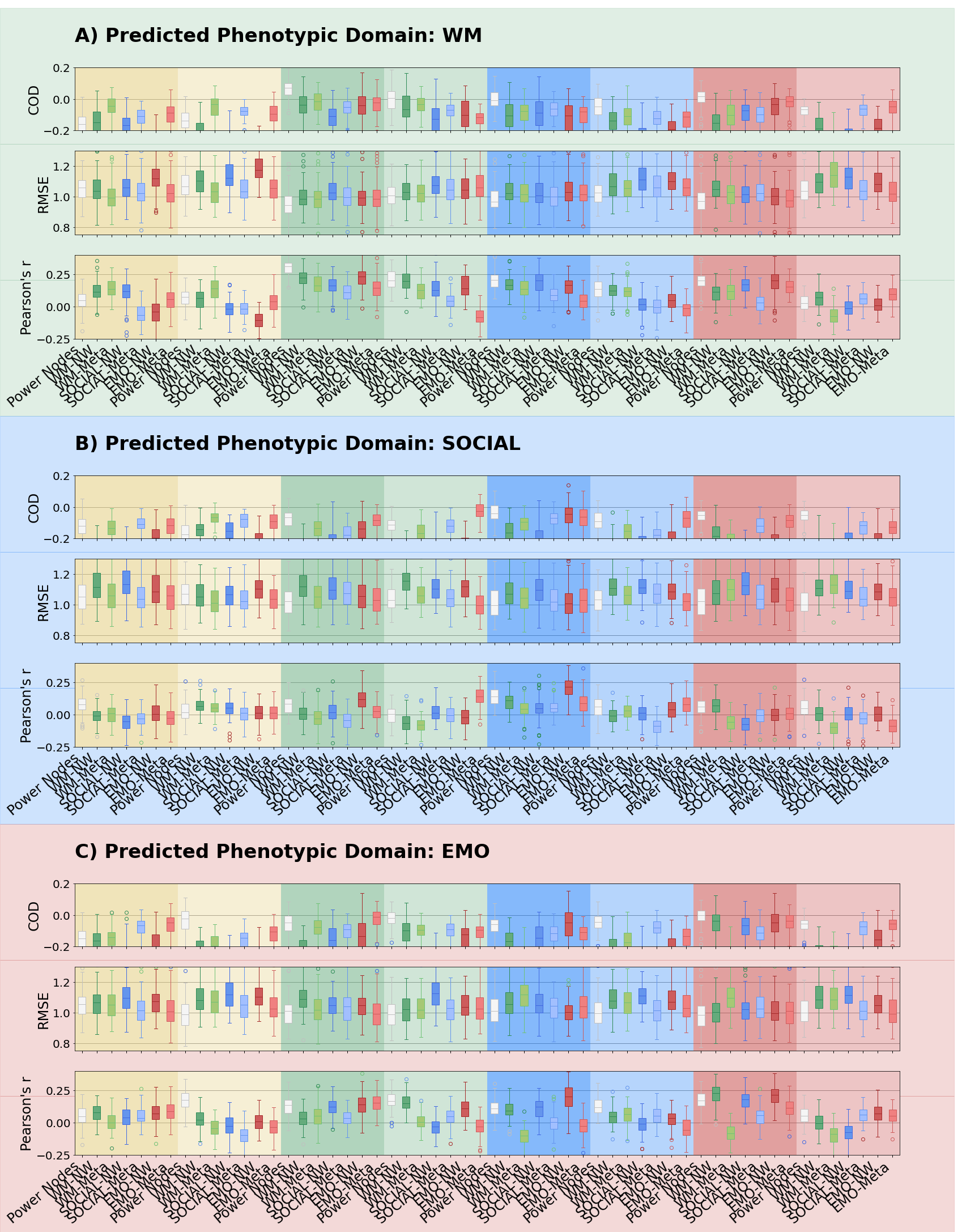  Figure S21) PLS 100 x leave-30%-out CV  Boxplots of the distribution of prediction accuracies from PLS 100 x leave-30%-out CV for WM, SOCIAL, and EMO domain, for coefficient of determination (COD) / model fit, RMSE and Pearson’s r. |
| --- |

| 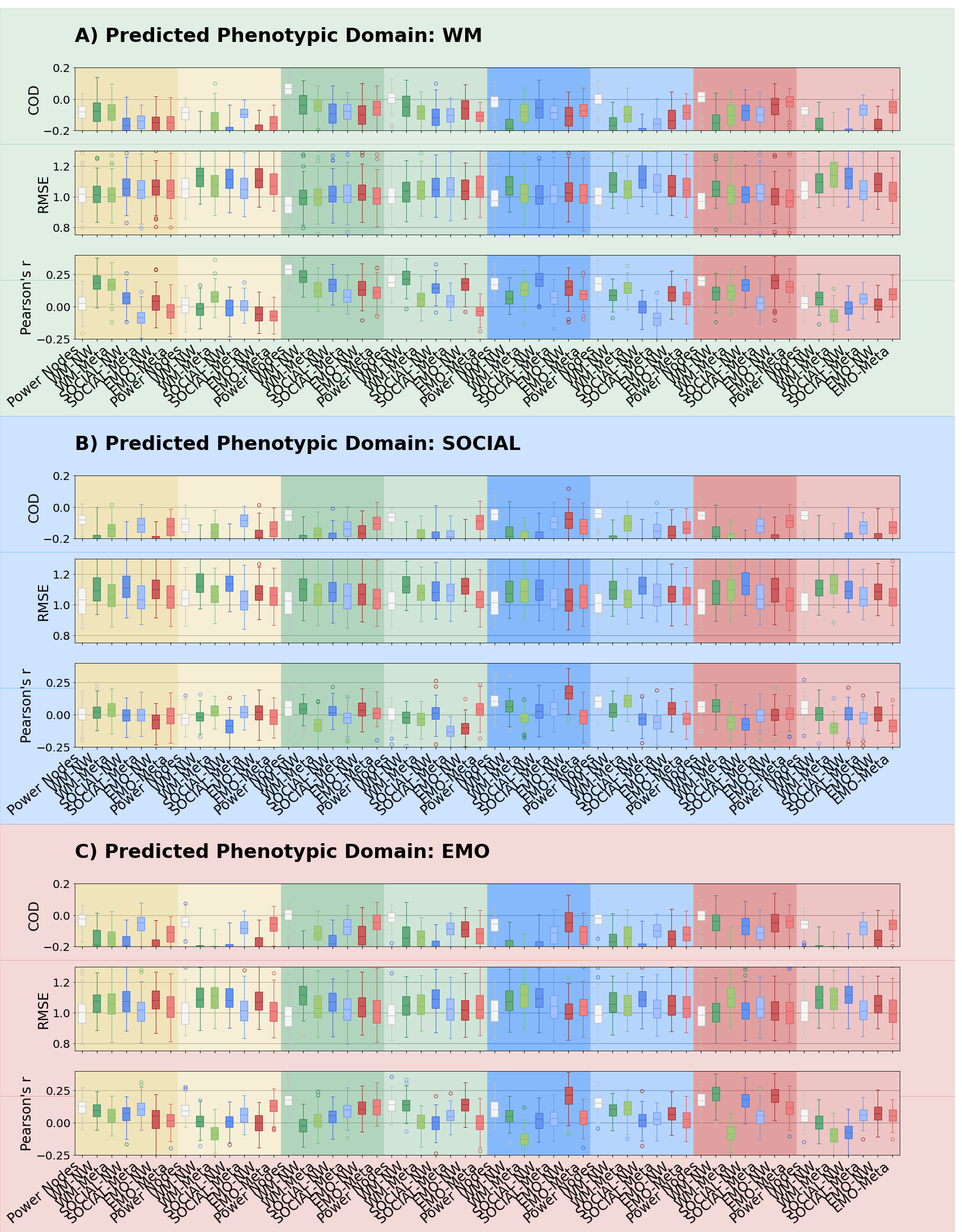  Figure S22) PLS 100 x leave-30%-out CV – trimmed time series  Boxplots of the distribution of prediction accuracies from PLS 100 x leave-30%-out CV – trimmed time series for WM, SOCIAL, and EMO domain, for coefficient of determination (COD) / model fit, RMSE and Pearson’s r. |
| --- |

| 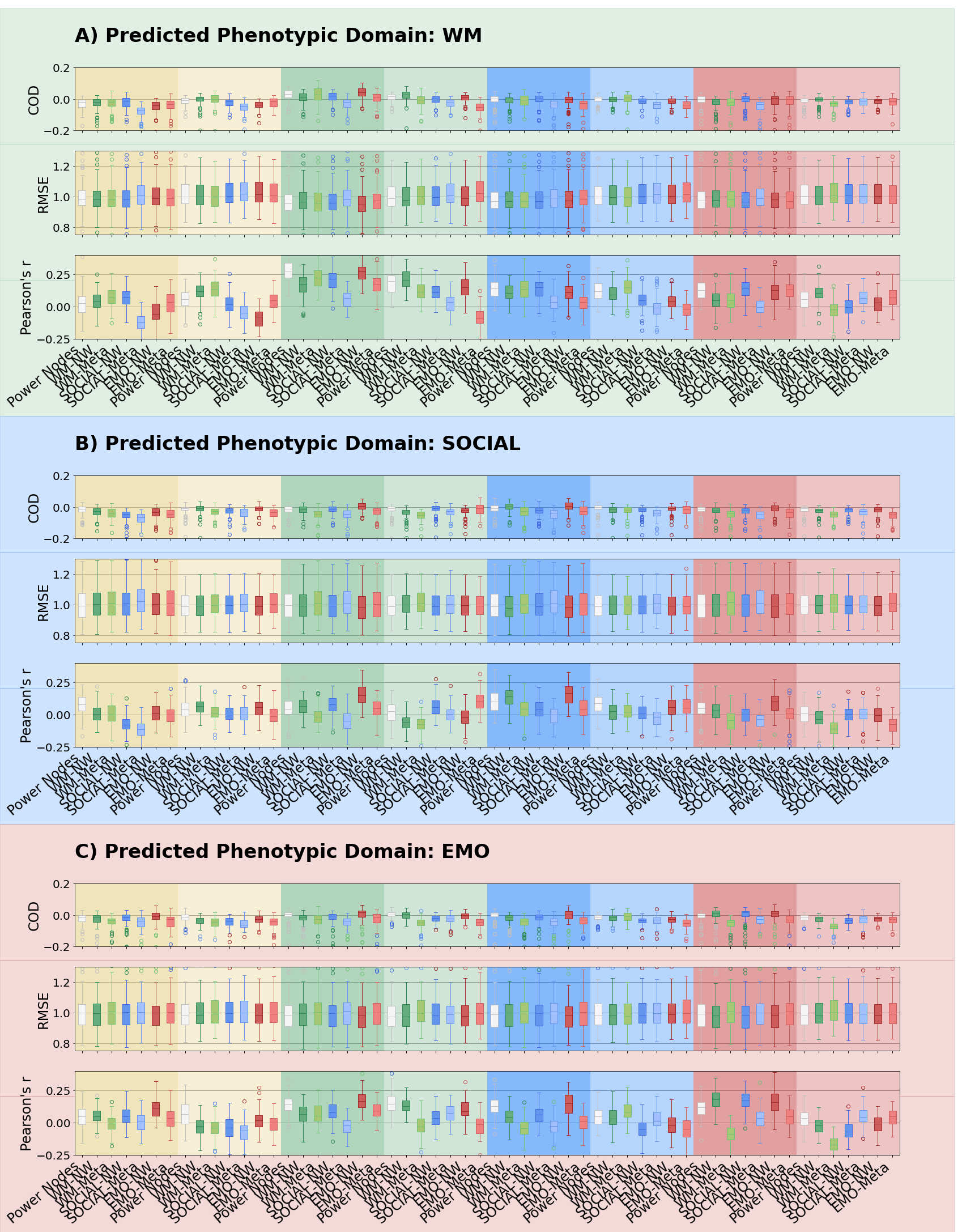  Figure S23) Random Forest 100 x leave-30%-out CV  Boxplots of the distribution of prediction accuracies from Random Forest 100 x leave-30%-out CV for WM, SOCIAL, and EMO domain, for coefficient of determination (COD) / model fit, RMSE and Pearson’s r. |
| --- |

| 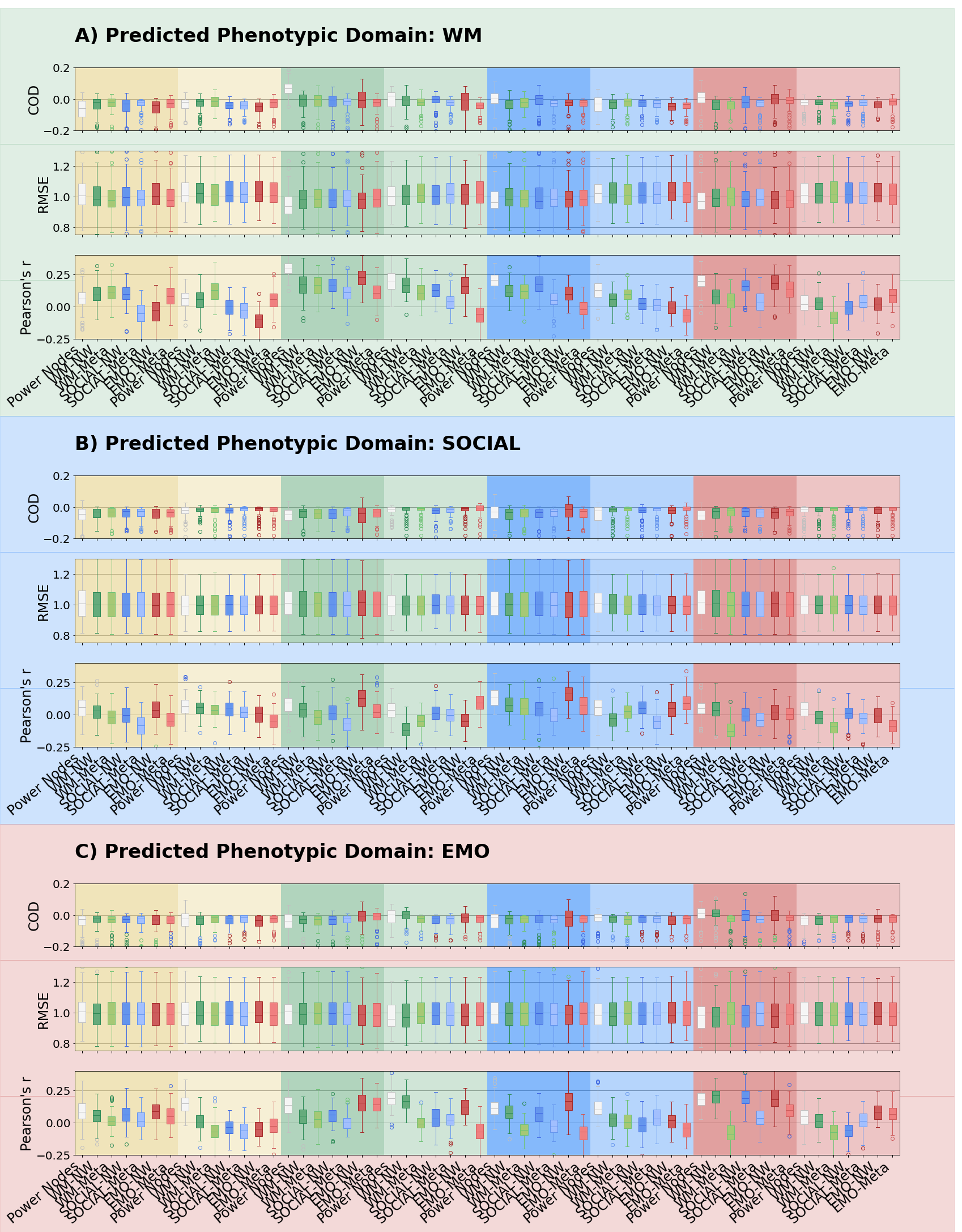  Figure S24) SVR – linear kernel - 100 x leave-30%-out CV  Boxplots of the distribution of prediction accuracies from SVR – linear kernel - 100 x leave-30%-out CV for WM, SOCIAL, and EMO domain, for coefficient of determination (COD) / model fit, RMSE and Pearson’s r. |
| --- |

| 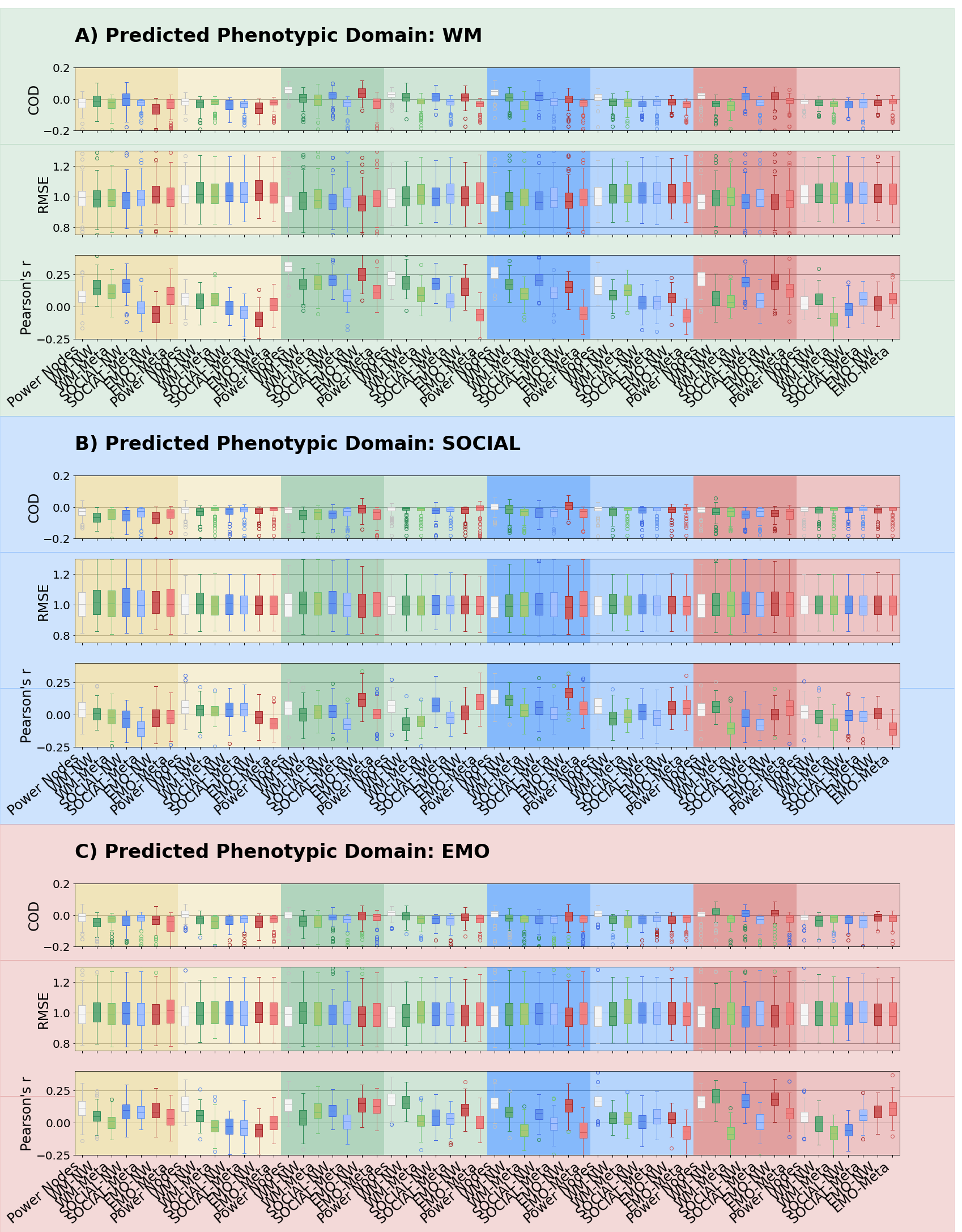  Figure S25) SVR – rbf kernel - 100 x leave-30%-out CV  Boxplots of the distribution of prediction accuracies from SVR – RBF kernel - 100 x leave-30%-out CV for WM, SOCIAL, and EMO domain, for coefficient of determination (COD) / model fit, RMSE and Pearson’s r. |
| --- |

| 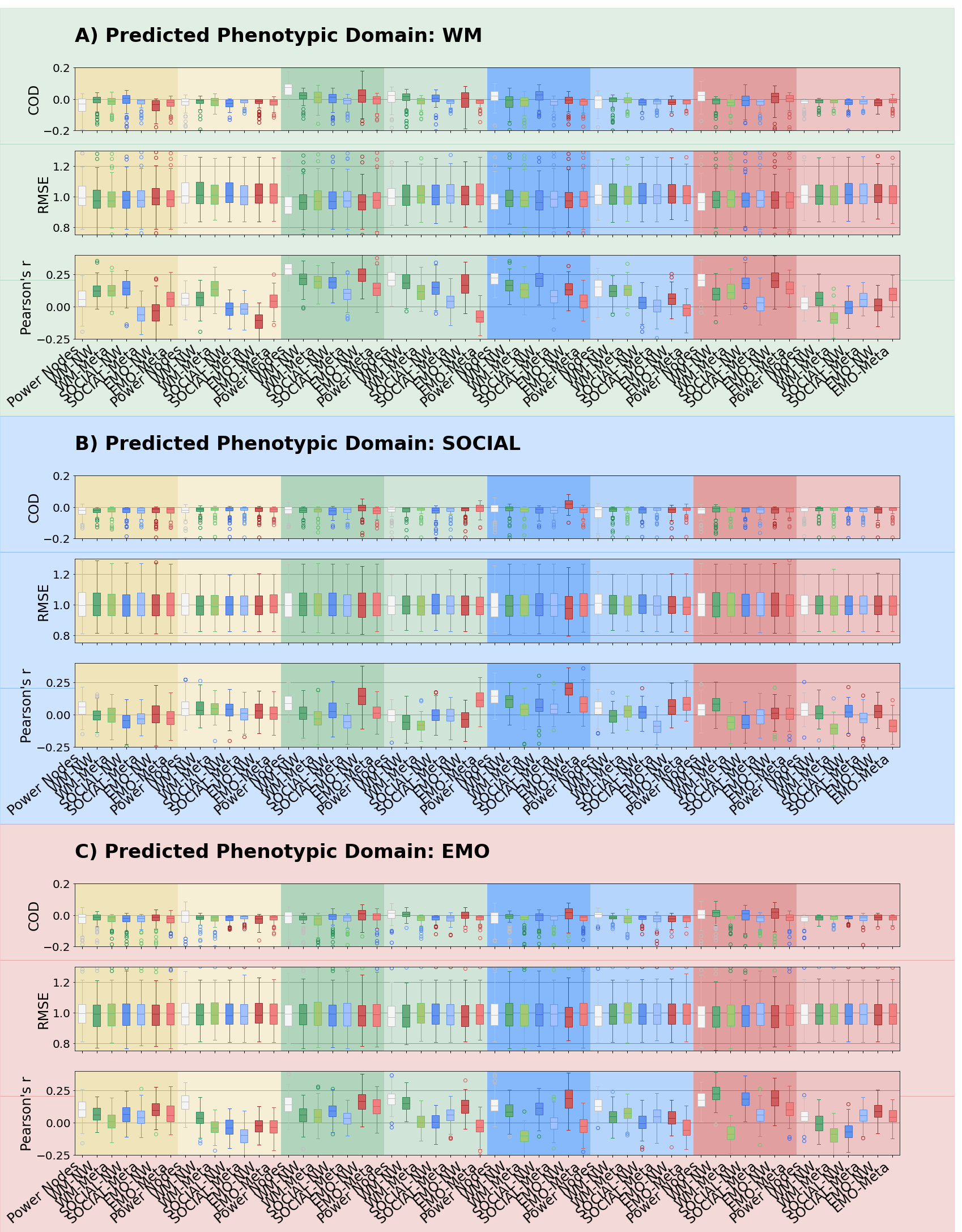  Figure S26) Kernel Ridge Regression - 100 x leave-30%-out CV  Boxplots of the distribution of prediction accuracies from Kernel Ridge Regression - 100 x leave-30%-out CV for WM, SOCIAL, and EMO domain, for coefficient of determination (COD) / model fit, RMSE and Pearson’s r. |
| --- |

| 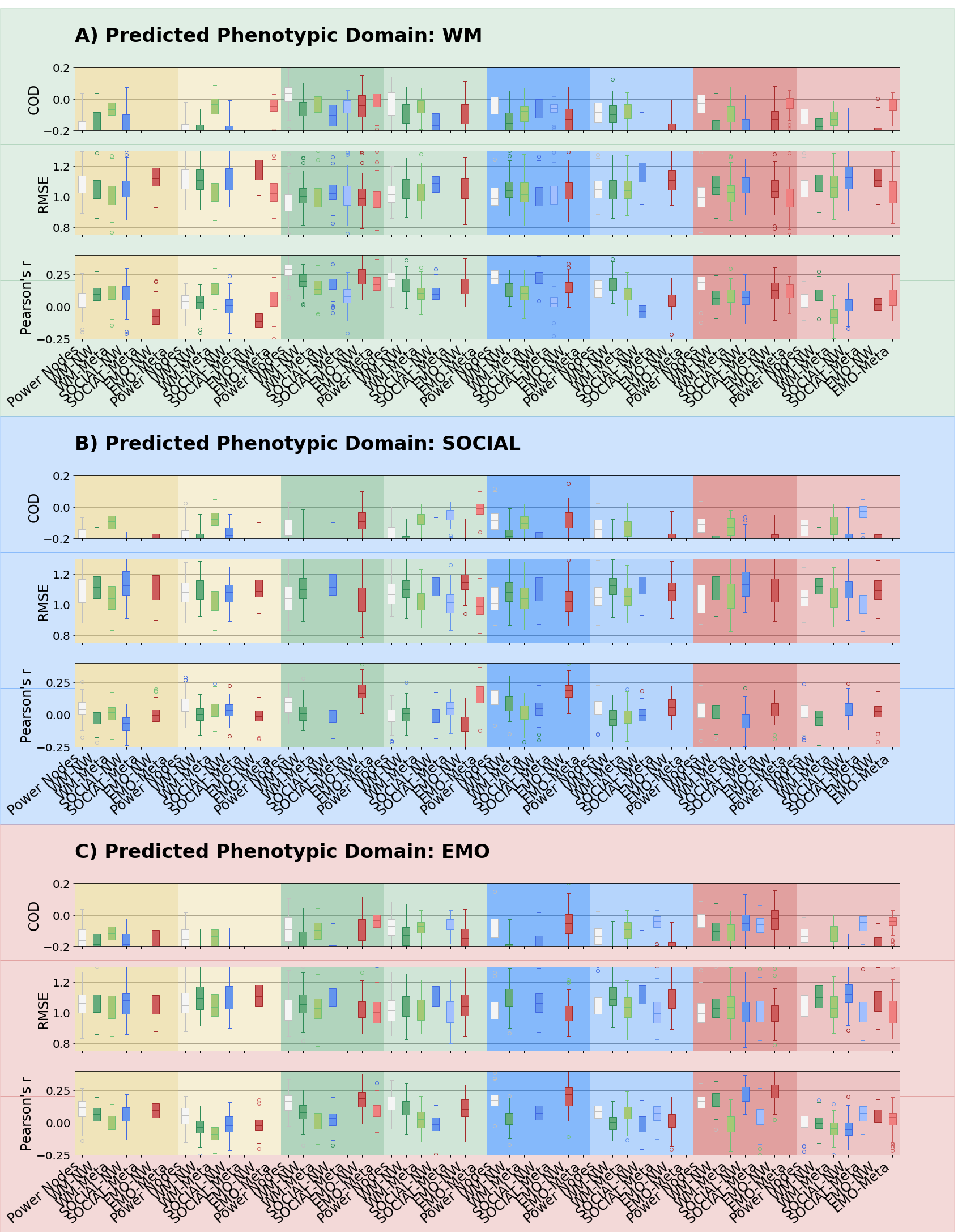  Figure S27) CBPM – with PLS - 100 x leave-30%-out CV  Boxplots of the distribution of prediction accuracies from CBPM – with PLS - 100 x leave-30%-out CV for WM, SOCIAL, and EMO domain, for coefficient of determination (COD) / model fit, RMSE and Pearson’s r. |
| --- |

| 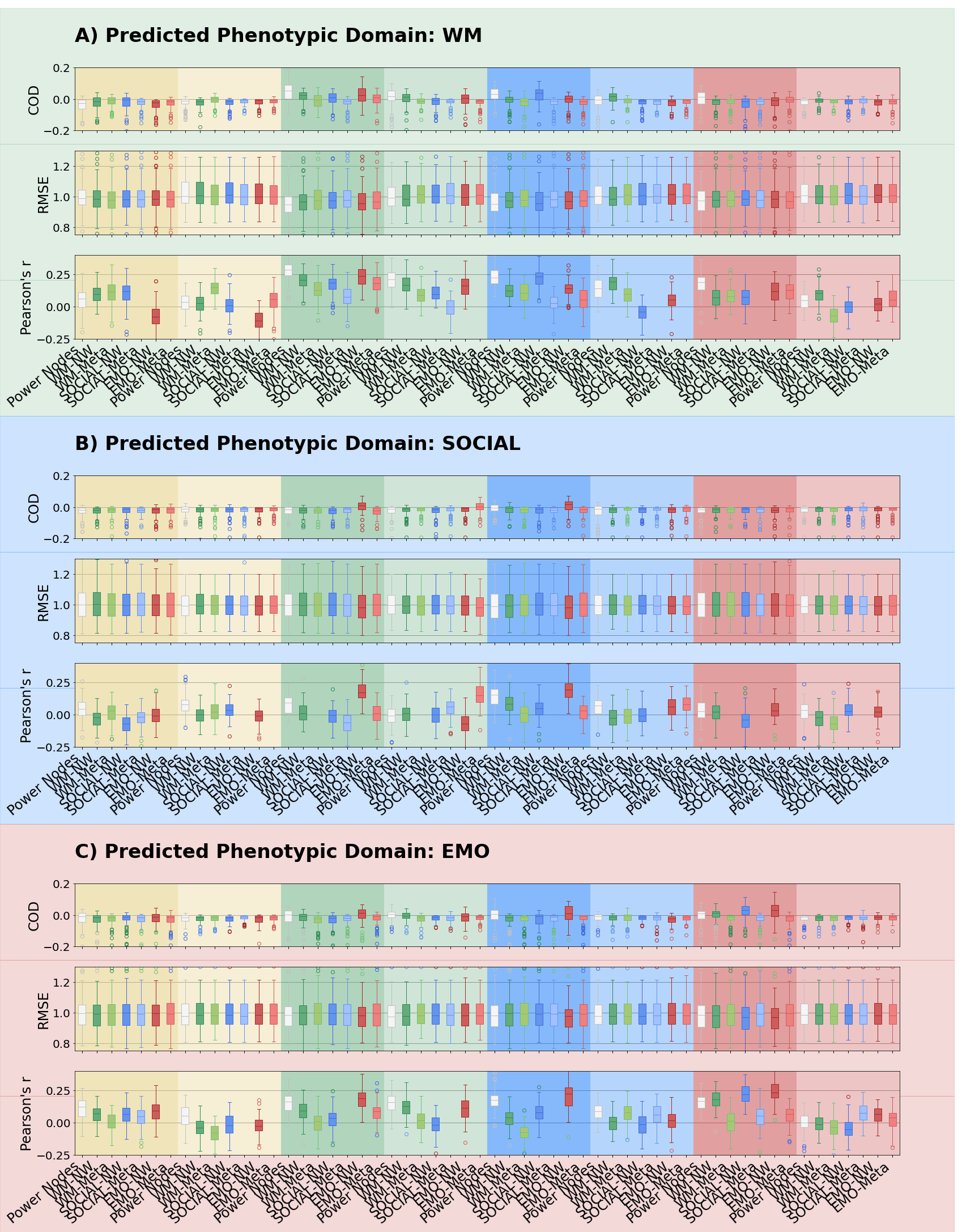  Figure S28) CBPM – with ridge regression - 100 x leave-30%-out CV  Boxplots of the distribution of prediction accuracies from CBPM – with ridge regression - 100 x leave-30%-out CV for WM, SOCIAL, and EMO domain, for coefficient of determination (COD) / model fit, RMSE and Pearson’s r. |
| --- |

| 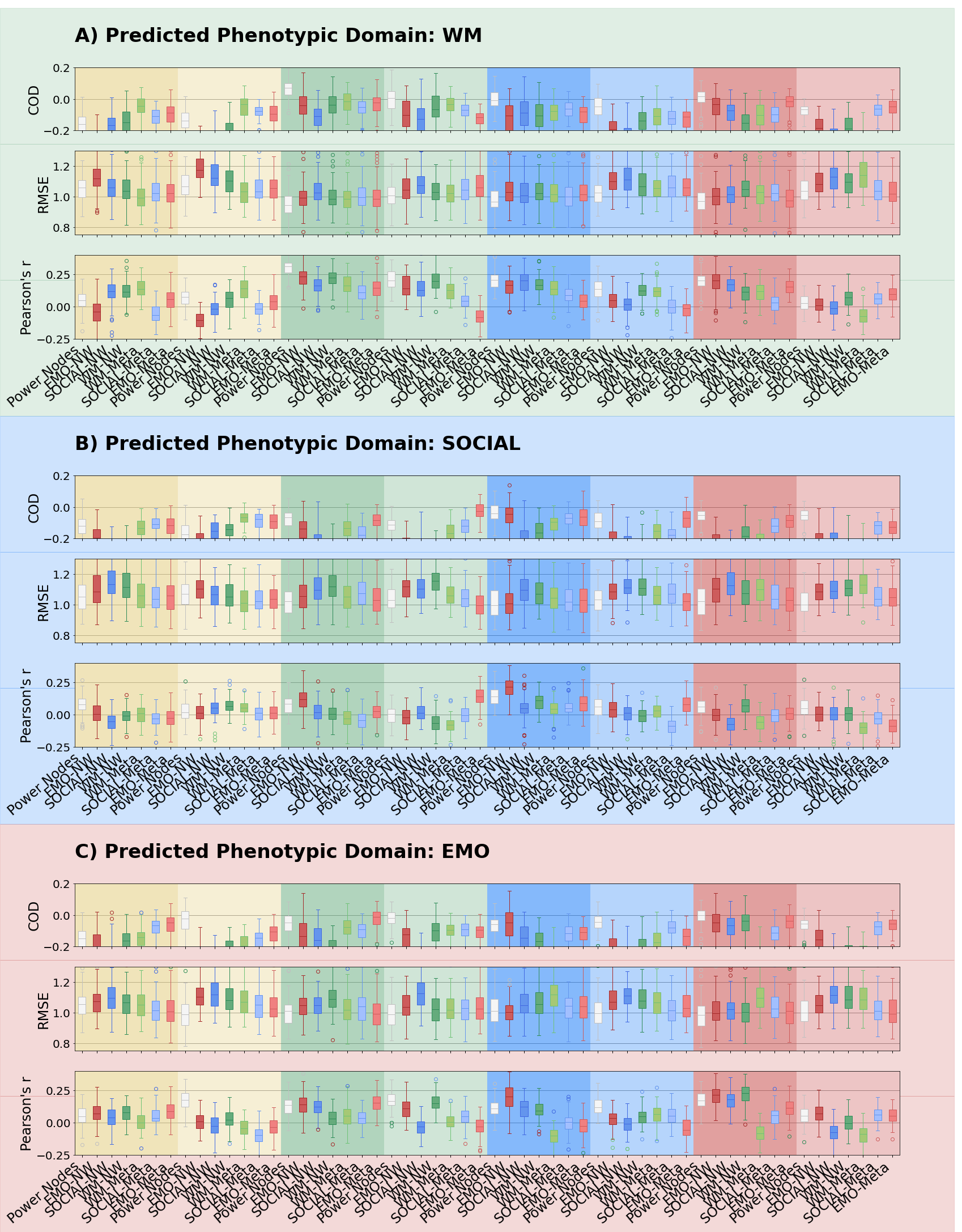  Figure S29. PLS 100 x leave-30%-out CV - sorted by network size  Boxplots of the distribution of prediction accuracies from PLS 100 x leave-30%-out CV for WM, SOCIAL, and EMO domain, for coefficient of determination (COD) / model fit, RMSE and Pearson’s r – sorted by network size from large to small: Power (264 nodes), EMO-NW (84 nodes), SOCIAL-NW (66 nodes), WM-NW (49 nodes), WM-meta-NW (19 nodes), SOCIAL-meta-NW (11 nodes) to EMO-meta-NW (10 nodes) |
| --- |

# Supplemental References

Babakhanyan, I., McKenna, B. S., Casaletto, K. B., Nowinski, C. J., & Heaton, R. K. (2018). National Institutes of Health Toolbox Emotion Battery for English- and Spanish-speaking adults: Normative data and factor-based summary scores. *Patient Related Outcome Measures*, *9*, 115–127. https://doi.org/10.2147/PROM.S151658

Barch, D. M., Burgess, G. C., Harms, M. P., Petersen, S. E., Schlaggar, B. L., Corbetta, M., Glasser, M. F., Curtiss, S., Dixit, S., Feldt, C., Nolan, D., Bryant, E., Hartley, T., Footer, O., Bjork, J. M., Poldrack, R., Smith, S., Johansen-Berg, H., Snyder, A. Z., … WU-Minn HCP Consortium. (2013). Function in the human connectome: Task-fMRI and individual differences in behavior. *NeuroImage*, *80*, 169–189. https://doi.org/10.1016/j.neuroimage.2013.05.033

Castelli, F., Happé, F., Frith, U., & Frith, C. (2000). Movement and Mind: A Functional Imaging Study of Perception and Interpretation of Complex Intentional Movement Patterns. *NeuroImage*, *12*(3), 314–325. https://doi.org/10.1006/nimg.2000.0612

*Cognition Measures*. (n.d.). Retrieved May 11, 2022, from https://www.healthmeasures.net/explore-measurement-systems/nih-toolbox/intro-to-nih-toolbox/cognition

*Emotion Measures*. (n.d.). Retrieved May 11, 2022, from https://www.healthmeasures.net/explore-measurement-systems/nih-toolbox/intro-to-nih-toolbox/emotion

Finn, E. S., Shen, X., Scheinost, D., Rosenberg, M. D., Huang, J., Chun, M. M., Papademetris, X., & Constable, R. T. (2015). Functional connectome fingerprinting: Identifying individuals using patterns of brain connectivity. *Nature Neuroscience*, *18*(11), 1664–1671. https://doi.org/10.1038/nn.4135

Gur, R. C., Richard, J., Hughett, P., Calkins, M. E., Macy, L., Bilker, W. B., Brensinger, C., & Gur, R. E. (2010). A cognitive neuroscience-based computerized battery for efficient measurement of individual differences: Standardization and initial construct validation. *Journal of Neuroscience Methods*, *187*(2), 254–262. https://doi.org/10.1016/j.jneumeth.2009.11.017

Gur, R. C., Sara, R., Hagendoorn, M., Marom, O., Hughett, P., Macy, L., Turner, T., Bajcsy, R., Posner, A., & Gur, R. E. (2002). A method for obtaining 3-dimensional facial expressions and its standardization for use in neurocognitive studies. *Journal of Neuroscience Methods*, *115*(2), 137–143. https://doi.org/10.1016/S0165-0270(02)00006-7

Hariri, A. R., Tessitore, A., Mattay, V. S., Fera, F., & Weinberger, D. R. (2002). The Amygdala Response to Emotional Stimuli: A Comparison of Faces and Scenes. *NeuroImage*, *17*(1), 317–323. https://doi.org/10.1006/nimg.2002.1179

Kogler, L., Müller, V. I., Werminghausen, E., Eickhoff, S. B., & Derntl, B. (2020). Do I feel or do I know? Neuroimaging meta-analyses on the multiple facets of empathy. *Cortex*, *129*, 341–355. https://doi.org/10.1016/j.cortex.2020.04.031

Müller, V. I., Cieslik, E. C., Laird, A. R., Fox, P. T., Radua, J., Mataix-Cols, D., Tench, C. R., Yarkoni, T., Nichols, T. E., Turkeltaub, P. E., Wager, T. D., & Eickhoff, S. B. (2018). Ten simple rules for neuroimaging meta-analysis. *Neuroscience & Biobehavioral Reviews*, *84*, 151–161. https://doi.org/10.1016/J.NEUBIOREV.2017.11.012

Müller, V. I., Höhner, Y., & Eickhoff, S. B. (2018). Influence of task instructions and stimuli on the neural network of face processing: An ALE meta-analysis. *Cortex*, *103*, 240–255. https://doi.org/10.1016/j.cortex.2018.03.011

Nadeau, C., & Bengio, Y. (1999). Inference for the generalization error. *Advances in Neural Information Processing Systems*, *12*.

Reid, A. T., Bzdok, D., Genon, S., Langner, R., Müller, V. I., Eickhoff, C. R., Hoffstaedter, F., Cieslik, E.-C., Fox, P. T., Laird, A. R., Amunts, K., Caspers, S., & Eickhoff, S. B. (2016). ANIMA: A data-sharing initiative for neuroimaging meta-analyses. *NeuroImage*, *124*, 1245–1253. https://doi.org/10.1016/j.neuroimage.2015.07.060

Rottschy, C., Langner, R., Dogan, I., Reetz, K., Laird, A. R., Schulz, J. B., Fox, P. T., & Eickhoff, S. B. (2012). Modelling neural correlates of working memory: A coordinate-based meta-analysis. *NeuroImage*, *60*(1), 830–846. https://doi.org/10.1016/j.neuroimage.2011.11.050

Salsman, J. M., Butt, Z., Pilkonis, P. A., Cyranowski, J. M., Zill, N., Hendrie, H. C., Kupst, M. J., Kelly, M. A. R., Bode, R. K., Choi, S. W., Lai, J.-S., Griffith, J. W., Stoney, C. M., Brouwers, P., Knox, S. S., & Cella, D. (2013). Emotion assessment using the NIH Toolbox. *Neurology*, *80*(11 Suppl 3), S76-86. https://doi.org/10.1212/WNL.0b013e3182872e11

Shen, X., Finn, E. S., Scheinost, D., Rosenberg, M. D., Chun, M. M., Papademetris, X., & Constable, R. T. (2017). Using connectome-based predictive modeling to predict individual behavior from brain connectivity. *Nature Protocols*, *12*(3), 506–518. https://doi.org/10.1038/nprot.2016.178

Wheatley, T., Milleville, S. C., & Martin, A. (2007). Understanding Animate Agents: Distinct Roles for the Social Network and Mirror System. *Psychological Science*, *18*(6), 469–474. https://doi.org/10.1111/j.1467-9280.2007.01923.x
